# Ocean connectedness, experiences, and stewardship: a qualitative study of American adults

**DOI:** 10.3389/fpsyg.2025.1657132

**Published:** 2026-01-05

**Authors:** Chris O’Halloran

**Affiliations:** Healthy Oceans, Healthy People, Santa Cruz, CA, United States

**Keywords:** blue spaces, ocean connectedness, positive emotions, pro-environmental behaviors, well-being

## Abstract

**Introduction:**

This qualitative study explores the various ways in which Americans experience the marine environment.

**Methods:**

To this end, thematic analysis was applied to analyze survey responses of a representative sample (*N* = 1,138) of U.S. adults, standardized for age, sex, and regional location. Two major vantage points for the analysis were what Americans think the ocean teaches them about life, as well as what they consider meaningful ocean experiences.

**Results:**

The results revealed that important themes related to ocean connectedness include well-being, positive emotions, recreational activities, encounters with marine creatures, bonding with friends and family, as well as spiritual and cultural connections.

**Discussion:**

These nuanced and rich insights can help to design impactful ocean conservation campaigns that, in turn, will foster deeper appreciation for the ocean’s life-sustaining role and may promote ocean stewardship.

## 1 Introduction

Covering nearly 71% of the Earth’s surface, the oceans (Atlantic, Arctic, Indian, Pacific, and Southern) connect, sustain, provide, inspire, and nurture all life on our planet (IPCC, 2019; Intergovernmental Oceanographic Commission, 2021). Ocean ecosystem health and human health and well-being are intricately interconnected (Fleming et al., 2015). The Ocean has served as a depository for excess atmospheric heat and carbon dioxide, and anthropogenic waste. By producing atmospheric oxygen through marine photosynthetic phytoplankton, the global Ocean supports life, as well as provides freshwater through the hydrological cycle and supplies seafood, an important source of protein for many coastal populations (IPCC, 2019). With a global population of over 8 billion people sustainable use of our precious and limited natural resources is of critical importance (Fleming et al., 2023). Ocean health is also a strong predictor of human health: available evidence suggests that human contact with safe natural environments is associated with numerous positive outcomes for both physical health (Lovell et al., 2018; O’Halloran and Silver, 2022) and psychological well-being (Capaldi et al., 2014; McMahan and Estes, 2015; O’Halloran and Silver, 2022).

However, with the advent of the Industrial Revolution, which necessitated massive burning of fossil fuels, the global Ocean came to function as a buffer absorbing massive amounts of carbon dioxide and 90% of the excess heat generated by greenhouse gases (IPCC, 2019; Friedlingstein et al., 2019; Fleming and Landrigan, 2024). This led to ocean acidification, coral bleaching, and a loss of marine biodiversity. Further anthropogenic impacts with a considerable negative impact on the health of marine ecosystems include plastic pollution, overfishing, destructive fishing practices like bottom trawling, agricultural runoff, chemical contamination, and the destruction of coastal habitats. Among dire consequences of human activities on the Ocean are rising sea surface temperatures, melting polar glaciers, sea level rise, harmful algal blooms, dead zones from depleted oxygen, and loss of marine biodiversity (Fleming et al., 2015).

To address the previously mentioned ocean problems, as well as to conserve and sustainably manage oceans, seas, and marine resources for a healthy and resilient marine environment, the United Nations declared the years 2021–2030 the Decade of Ocean Science for Sustainable Development (United Nations, 2020). In addition, ocean conservation and sustainable use of oceans, seas, and marine resources was formulated as Sustainable Development Goal 14 of the United Nations (United Nations, 2020; Intergovernmental Oceanographic Commission, 2021). Along with international and government regulations, small individual actions–such as reducing plastic use, sustainable (sea) food choices, and lower carbon footprints (e.g., less car and air travel, reducing energy consumption)–can also yield substantial impacts (O’Halloran, 2024; Noujua et al., 2022; McKinley and Fletcher, 2010; Whitmarsh and O’Neill, 2010). For instance, approximately one-fifth of energy-related greenhouse gas (GHG) emissions in the United States are reported to be generated by individual households (US Energy Information Administration, 2019).

A strong predictor of pro-environmental behaviors (PEBs) is human contact with safe natural environments (Arendt and Matthes, 2016). Time spent in nature can make environmental issues feel less abstract and more personally relevant by helping people to see themselves as part of nature rather than separate from it. There is also evidence to suggest that PEBs can be maximized by developing a psychological or emotional bond with nature (Capaldi et al., 2014). This suggests the need for an integrated approach to environmental sustainability that would seek to enhance people’s awareness about the interconnectedness of environmental health, on the one hand, and human health and well-being, on the other hand (Depledge et al., 2019). This approach would emphasize both protection of ecosystems and support of human well-being, recognizing that healthy environments and healthy people are mutually reinforcing.

### 1.1 Nature connectedness (terrestrial)

Connectedness to nature is defined as “the extent to which an individual includes nature within his/her cognitive representation of self” (Schultz, 2000, p. 67). In empirical research, nature connectedness is measured using several psychometric scales, such as Connectedness to Nature Scale (Mayer and Frantz, 2004) and the Nature Relatedness Scale (Nisbet and Zelenski, 2013). Several theoretical frameworks explaining nature connectedness have been proposed. According to the biophilia hypothesis formulated by Wilson (1986), owing to evolutionary connections of resources and genetic factors, humans possess an innate affinity with the natural world. In other words, human preference for natural environments is underpinned by the fact that such environments historically provided humans with essential resources such as food, shelter, and comfort necessary for survival (Kellert, 1993). Furthermore, these ingrained aesthetic and emotional preferences for nature are assumed to trigger cognitive and physiological responses that further reinforce beneficial behaviors, such as social and resource cooperation.

Furthermore, another pertinent theory–the connectedness to nature theory–posits that, driven by the sense of connection to the natural world, individuals with a strong bond with nature are more likely to engage in PEBs that protect and sustain the natural environment (Mayer and Frantz, 2004). Such behaviors include reducing waste, eating plant-based diets or sustainable seafood, reducing energy consumption, and supporting conservation initiatives (Noujua et al., 2022; O’Halloran, 2025). In support of this theory, Martin et al. (2020) found that frequent contact with nature, such as weekly visits or watching nature documentaries, was associated with well-being and PEBs. Collectively, these frameworks suggest that feeling connected to nature is not only a psychological state but a potential driver of everyday choices that benefit the environment.

### 1.2 Ocean connectedness

Most previous research on nature connectedness has focused on terrestrial environments, rather than on marine environments. However, in the context of recent threats to ocean health, scholarly interest in ocean connectedness, broadly understood as the affinity people feel toward the marine environment, has considerably increased (O’Halloran, 2025; Noujua et al., 2022; White et al., 2021; Spence et al., 2012; Tonge et al., 2015). Current research findings suggest that ocean connectedness is influenced by culture, recreational activities, meaningful marine encounters, emotions, well-being, and spirituality (O’Halloran, 2025; McKinley et al., 2023; Noujua et al., 2022; Buchan, 2021). Furthermore, this connection with the marine environment was documented to promote PEBs and foster a deeper appreciation for the ocean’s life-sustaining role (O’Halloran, 2025; Noujua et al., 2022). For instance, Noujua et al. (2022) found that emotional connections to the ocean can influence sustainable behaviors, such as choosing eco-friendly alternatives and decreasing plastic consumption. In another relevant study, Pahl et al. (2017) found that an emotional affinity with the ocean leads to PEBs. Taking this research further, in a systematic review on ocean literacy and behavior change, Stoll-Kleemann (2019) concluded that emotional connections are important to incorporate into educational initiatives to promote PEBs. While Buchan (2021) found that emotional connections, marine identity, and pro-environmental behavior can effectively inspire ocean stewardship. McKinley et al. (2023), in their model of ocean literacy, identified emotional connection (emoceans) as one of ten dimensions that influence ocean-related behaviors and PEBs.

### 1.3 Research gap and contribution

A large body of work has associated nature connectedness to well-being and PEBs, but ocean connectedness is comparatively under examined especially in a qualitative manner. Much of the existing literature quantifies emotions, environmental identity, or PEBs without exploring individual’s marine experiences, positive and negative, that may motivate ocean stewardship. A qualitative approach captures participants’ experiences in their own words in a nuanced way that standardized measures may miss. This study thematically analyzed two open-response questions, from a larger study on ocean connectedness, to inform messaging for conservation campaigns and initiatives as well as to generate hypotheses for subsequent quantitative studies. The phrasing of these prompts likely encouraged respondents to offer reflective, personally meaningful, and often positive narratives about the ocean.

The present qualitative study was part of a larger study investigating the association between ocean connectedness and PEBs. That quantitative study found that ocean connectedness, environmental identity, positive emotions (e.g., happiness, gratitude, awe, and joy), and engagement in ocean activities (e.g., surfing, fishing) were strongly associated with increased PEBs (O’Halloran, 2025). The goal of the present study is to explore how Americans experience the marine environment to obtain a more nuanced understanding of ocean connectedness to help design more effective well-being and conservation interventions. To this end, thematic analysis is applied to analyze the participants’ responses to two survey questions–specifically, on what the ocean teaches us about life and meaningful marine experiences. The study describes how American adults experience the ocean and identifies themes with the potential to support well-being interventions and ocean conservation stewardship.

## 2 Materials and methods

### 2.1 Survey

A qualitative research design was used to explore the concept of ocean connectedness among a representative sample of 1,138 American respondents. The data were collected using a cross-sectional anonymous online survey by Survey Monkey Audience^1^. Survey Monkey is operated by Symphony Technology Group which incentivizes survey respondents with gift card credits, sweepstake entries, and donations to charities. The self-reported participant data included demographic information (e.g., sex, age, race, educational level), as well as the participants’ responses on ocean connectedness, pro-environmental behaviors, environmental identity, emotions, and ocean/beach related activities.

This study was administered July 31–August 1, 2024, with respondents answering 12 quantitative questions and two open-ended questions. The quantitative results have been recently published (O’Halloran, 2025). This paper focuses on the results of the two qualitative questions that inquired about the participants’ perceptions of what the ocean teaches about life and meaningful ocean experiences: (1) “In your opinion, what does the ocean teach us about life?” and (2) “Describe a meaningful experience or encounter you had in the ocean, on the beach, or in a marine environment.” Responses ranged from one-word answers to several sentences detailing meaningful experiences. Although response length was unconstrained, framing the prompt around “meaningful” experiences likely encouraged reflective, salient, and predominantly positive accounts.

### 2.2 Data analysis

Data analysis unfolded in several steps. First, a word count frequency was examined to identify the most frequently used words (i.e., everything, good, beautiful, teaches, swimming, beach…) and visualized with a word cloud to identify dominant themes. Then a thematic analysis approach was used to identify themes in the data. The analysis followed Braun and Clarke’s (2006) six-step process: (i) familiarization with the responses; (ii) coding the responses; (iii) reviewing codes and (iv) grouping them into themes; (v) reviewing themes to ensure accuracy, and (vi) description the final themes with clarifying quotes. This thematic approach allowed themes to emerge from the data without imposing preconceived categories. Coding was conducted by a single researcher who engaged in an iterative, interpretive process. The full dataset was coded three times in independent passes to enhance within-coder consistency and to refine the developing codebook. Across passes, the researcher systematically reviewed and refined code and theme definitions in the codebook. When potential ambiguities were identified, for example, responses that partially fit more than one theme, definitions were clarified and the codebook updated before the final pass, and no unresolved inconsistencies in theme application were detected. Responses were coded into one or more themes. One-word responses were included if they fit into a theme (e.g., connectedness [theme: interconnectedness], good [theme: life is good]). Ambiguous one-word responses (e.g., trade life, g, occasionally) were excluded.

Thematic mentions of meaningful ocean experiences and encounters (Wildlife; Well-being; Adventure and Recreation; Friends, Family and Social Connections; Good/Great; Stewardship; One/Spirituality) were coded as binary outcomes (0/1). For each theme, bivariate binomial generalized linear models with a log link were estimated to screen potential predictors; variables showing evidence of association (*p* < 0.05) advanced to multivariable analysis. The final models used a binomial family with a log link to estimate risk ratios (RRs) and 95% confidence intervals (CIs). Predictors included gender, ethnicity, age categories, and U.S. Census geographic regions. Models were fit in Stata using the command glm dependent variable independent variables, family(binomial) link(log) eform.

### 2.3 Study participants

Eligibility criteria were as follows: (i) U.S. residence; (ii) age at least 18 years old; (iii) English reading fluency; and (iv) internet access to complete the survey. Before starting the survey, all participants provided informed consent electronically through Survey Monkey Audience. Among candidate respondents, 28 (2.3%) did not consent and were excluded from the study. A total of 1,138 individuals completed the survey (response rate: 93%). The respondents’ demographic characteristics are summarized in Table 1. Most of the participants were aged 45–60 years old (29.5%), followed by those aged 30–44 years (26.6%), over 60 years (24.5%), and 18–29 years old (21.8%). Over half of the respondents were female (52.6%). About two thirds of the sample were White (67.8%) and slightly over half (51.1%) of the respondents had a college degree.

**TABLE 1:** Demographic characteristics of participants (*N* = 1,138).

Characteristic	*n*	%
**Sex**		
Female	613	53.87
Male	525	46.13
**Age range (years)**		
18–29	248	21.27
30–44	303	25.99
45–60	336	28.83
>60	279	23.93
**Ethnicity^a^**		
White	772	67.84
Asian	157	13.80
Latino	105	9.23
Native American/Alaskan Native	102	8.96
Black	96	8.44
Multi-racial	61	5.36
Native Hawaiian/Pacific Islander	31	2.72
**Education**		
College graduate	582	51.14
No college degree	556	48.86

^a^Participants could select more than one ethnicity.

## 3 Results

### 3.1 What the ocean teaches us about life?

Thematic analysis of the data identified important themes in the qualitative replies of the respondents. The seven themes, their descriptions, and sample responses to the question “What does the ocean teaches us about life?” are listed in Table 2. Almost two thirds (62%) of the respondents provided extended answers (e.g., phrases and sentences) to this question, while the remaining participants provided one-word answers (e.g., good, great, amazing). Participant responses were categorized as single theme or multiple themes. For example, this participant comment (e.g., “The ocean connects to all of us in some way. I find it extremely calming and beautiful. I love to sit and listen to the waves. Looking out across the sand to the water, heals my soul.”) was categorized into two themes: well-being and interconnectedness. One-word responses (e.g., “good,” “great,” “amazing,” “calmness,” “beauty”) were coded into themes when the meaning was clear (e.g., “good” in “Life is Good/Great”), while ambiguous or uninterpretable single words (e.g., “g,” “trade life”) were excluded from the thematic analysis. For this question, 22% of participants provided a one word response. Additionally, 5% of respondents reported that the ocean did not teach them anything about life.

**TABLE 2:** Themes in responses to “What does the ocean teach us about life?”

Theme	Description	Example quote	Percentage
Ocean as a source of well-being, calm, love, and aesthetic appreciation	The ocean as a source of peace, relaxation, and appreciation for the beauty of nature	“The ocean connects to all of us in some way. I find it extremely calming and beautiful.”	11%
Interconnectedness	Recognition of the interconnectedness of all living things and ecosystems	“The diversity but interconnectedness of all life on this planet.”	10%
Stewardship	Emphasis on human responsibility to protect and respect the ocean and its ecosystems	“We all need to work together to protect it.”	10%
Life is good/great	Gratitude that life is good or great	“Nature is good,” “good experiences,” “that life is good, great.”	6%
Resilience, adaptability	Lessons about change, adaptability, and going with the flow	“Oceans tide teaches us to embrace change and go with the flow.”	4%
Sustains us	The ocean’s role in sustaining all life and supporting ecosystems essential for planetary health	“It brings life to the people of this planet.”	4%
Teaches us about nature	Appreciation for the ocean’s creatures, vastness, biodiversity, and ecological systems	“Reminds us of the diversity of life on earth.”	3.5%

The most frequent theme, mentioned by 123 respondents (11%), described the ocean as a source of well-being that provides beauty, calmness, and peace. For instance, “It [the ocean] teaches us that life is beautiful and that we should take it easy,” “The ocean connects to all of us in some way. I find it extremely calming and beautiful. I love to sit and listen to the waves. Looking out across the sand to the water, heals my soul.” Responses in this theme also described the ocean as a source of relaxation (“To relax and go with the flow”) and beauty (“It teaches us that there is vast beauty in the world and sometimes things are loud and rough and irregular but also things can be calm, quiet and beautiful”). Participants frequently described the ocean as a source of psychological well-being, but in several distinct ways. Many emphasized the ocean as a place to “reset,” reduce stress, and “breathe again” when life felt overwhelming. Others highlighted love and emotional closeness, expressing affection for the ocean and for time shared with family, friends, or partners at the beach or in the ocean. The ocean was a significant place for deepened relationships and lasting memories. Additionally, participants described aesthetic appreciation through the beauty of ocean waves, light, color, and sounds. The ocean was described as “breathtaking,” “awe-inspiring,” or “magical,” often linking these sensory experiences to feelings of gratitude and perspective on life. While the emotions of calm, love, and aesthetic appreciation can be distinguished conceptually and may have different psychological consequences, they commonly appeared together in individual narratives. For example, feeling calmer while watching a beautiful sunset with someone they love. For this reason, these emotions are presented as a single overarching theme that captures multiple, related pathways through which the ocean supports emotional and relational well-being.

The second theme that emerged in the analysis of the respondents’ answers to the first question was the perception of all life as interconnected (*N* = 115; 10%). For instance, “Everything ebbs and flows, is connected,” “It’s important for us to coexist with the ocean because it’s so connected with all life on earth.” These narratives framed the ocean as life sustaining for the planet, supporting climate regulation, food webs, and human survival. Several respondents also linked this sense of interconnection to responsibility, noting that “we are part of the ocean, not above it” and that harming the ocean ultimately harms human communities. In this way, the theme reflected a relational way of thinking in which the ocean, humans, and other ocean species are intricately connected in the web of life.

The third theme, which emerged in the responses of 115 (10%) participants, concerned ocean stewardship, understood here as protecting and respecting the ocean. For instance, one respondent wrote that “It [the ocean] teaches us that there is more than what we see on the surface. There is beauty everywhere and we all need to work together to protect it.” A similar point was made by another participant: “It teaches us everything about life. Its origins, the diversity, and how we need to protect it to protect ourselves.” The theme of protecting the ocean also emerged in other responses, such as “It teaches us to care and protect something bigger than ourselves for a greater purpose” and “God created all environments and we must be good stewards of them all.” Collectively, these narratives suggest that for some participants, ocean connectedness includes an ethical dimension, in which care for the ocean is viewed as both a moral responsibility and a way to protect humanity.

The fourth theme, which was coded as “Life is Great/Good,” emerged in the responses of 66 (6%) participants who commented on the general positive effect the ocean (e.g., “Nature is good,” “good experiences,” “that life is good”). For some respondents the ocean reminds them that “life is beautiful” or that “there is still good in the world.” In many of these brief but powerful statements, the ocean was a place for joy, appreciation, and gratitude. Being in, on, or near the ocean was a reminder that things are “okay” or “better than we think.” Even when participants did not describe specific emotions such as calm, aesthetic appreciation, or awe their comments suggested that simply being by the ocean can reinforce a more optimistic outlook on life.

The next theme, mentioned by 47 (4%) respondents, concerned the perception that the ocean teaches us about resilience, adaptability, change, and going with the flow. For instance, one respondent highlighted that the ocean teaches us to be responsible: “There are two stories/crises with the ocean, those being coral death and the water level rising. Both may teach how life can change in the future based on what you do in the past.” Several other respondents highlighted adaptability: “Oceans tide teaches us to embrace change and go with the flow”; “You can either surf the wave or go under, but you cannot stop the waves.” One more participant provided a detailed account of how the ocean teaches us to go with the flow:

The ocean teaches us about the vastness and mystery of life. It reminds us of our smallness in the grand scheme of things and the importance of humility. The constant ebb and flow of tides symbolize the ups and downs we experience, illustrating the importance of resilience and adaptation. The ocean’s depth and diversity highlight the beauty of exploration and the value of embracing diversity. Its interconnected ecosystems show how everything in life is interdependent, teaching us the significance of balance and harmony.

The sixth theme in the analysis, mentioned by 46 (4%) respondents, was related to sustainability. Here, the participants mentioned how the ocean sustains us (e.g., “it gives life”; “it brings life to the people of this planet,” “the ocean is the earth’s heart beat”; “it’s literally the circle of life. We would die without it”). Within this theme, several respondents highlighted that individuals need to take action to preserve and protect the ocean: “It is part of our earth, and we need it in order to sustain life. It is vast, but it cannot clean itself, and if we abuse it, eventually we all pay the price”; “The “health” of the ocean reflects the general health of our environment.”

The seventh theme mentioned by 40 (3.5%) people, focused on the marine ecosystems, biodiversity, and learning about marine creatures. For example, “Reminds us of the diversity of life on earth,” and “Every organism is born, and every organism dies one way or another.” These responses often blended curiosity with respect. Participants described being fascinated by “how many species we still don’t know,” noticing “different fish, plants, and animals all in one place,” and recognizing that “the ocean is home for so many living things.” Even when brief, their comments described the ocean as a teacher of basic ecological principles, including biodiversity, food webs, and life cycles, and suggested that encounters with marine ecosystems can help some individuals see the planet as more complex, fragile, and alive than they had previously realized.

In addition, less frequently mentioned themes in response to “what does the ocean teach us about life” included power, balance, empathy for animals, fragility, exploration, and spirituality. Although these themes were not included in the main thematic table, they were present in participants’ narratives and point to additional ways in which the ocean is understood as a powerful, dynamic, and morally instructive presence in people’s lives.

### 3.2 Meaningful experiences and encounters in marine environments

Regarding the second question that inquired about meaningful ocean and beach experiences of the participants, almost two thirds (65%) elaborated with phrases or sentences, while 17% of respondents provided one-word answers (e.g., good, great, amazing, one). The results of the thematic analysis suggested seven general themes in the responses (see Table 3).

**TABLE 3:** Themes in response to meaningful ocean experiences.

Theme	Description	Example quote(s)	Percentage
Wildlife encounters/sea creatures	Memorable interactions with marine wildlife such as dolphins, stingrays, sea turtles, sea lions, fish, seabirds, whales, orcas, and sharks. These encounters are awe-inspiring and often shared with others.	“Walking on the beach with my family at sunset and seeing a pod of dolphins jumping in and out of the water”; “Seeing a shark in Bermuda.”	22%
Well-being, calm, aesthetic appreciation	The ocean is described as a calming, grounding place that provides tranquility, stress relief, and a sense of well-being. Many respondents express positive sentiments about marine experiences, finding peace, beauty, and rejuvenation in the ocean’s sights and sounds. Watching sunsets and the vastness of the sea are described as especially meaningful.	“My peaceful place was always to sit on a bluff above the ocean, breathe the air, listen to the waves and feel at one with the earth”; “Walking on the beach next to the ocean reminds me how small a part I am and what power the ocean has. I find it relaxing and comforting”; “When the world seems so harsh or I am stressed about something, the ocean consumes my senses and makes me feel better”; “The most beautiful sunsets are near water”; “Watching the sunset from a quiet beach… I felt a profound sense of peace and connection with nature.”	16%
Adventure and recreation	Engagement in marine activities such as snorkeling, scuba diving, kayaking, tide-pool exploration, and body surfing.	“Swimming with my children in the Gulf of Mexico!”; “I kayaked surrounded by sea lions in Cabo San Lucas. They were interacting with us and playing with us”; “Swimming with dolphins in the Gulf of Mexico”; “While snorkeling, seeing the wildlife under the surface is always meaningful to me”; “Once when I was kayaking in the Atlantic Ocean a huge sea turtle just popped its head up. It was so cool.”	14%
Family, friend, and social connections	The ocean as a place for bonding with family and friends and as a romantic setting, including proposals, weddings, and romantic walks with loved ones.	“Connecting and bonding with my kids and family while at the beach”; “I had a bonfire with friends at the beach, and the wind and the smell of the ocean and the feel of the sand reminded me that nature can help connect me with my physical being”; “We walked to Haystack Rock and ate some locally sourced clam chowder, we saw lots of marine life and just relaxed. At the bonfire, we talked about all our favorite memories of our family”; “I proposed to my wife in the ocean.”	13%
Good/great	The general ocean experience is reported as good or great.	“Great”; “Good”; “Nice”; “It’s fun”; “It was fantastic.”	15%
Stewardship, environmental responsibility, and respect	Experiences related to protecting the marine environment, such as beach cleanups, rescuing wildlife, and appreciating the importance of a clean ocean. Acts of stewardship and witnessing others help marine life are highlighted.	“I helped a crab back to the water”; “I saved a starfish from dying”; “We helped untangle a seabird stuck in fishing line”; “I remember being at a beach with a dolphin washed up on shore and I saw a lot of people help get it back into the ocean”; “We found a few washed up fishes… decided we should put them back even though they looked dead. They weren’t dead, just exhausted. They perked right up and swam away.”	7%
One, oneness, spiritual connection	The ocean as a source of feeling one with nature, spiritual healing, and connection. Respondents describe the ocean as a place for emotional and spiritual cleansing, gratitude, and religious experiences.	“One”; “An out-of-body-like oneness with it”; “Whenever I am near the ocean, I feel we are all a part of the bigger universe. The sound of the waves relaxes me”; “The ocean has always been my element. In my darkest sadness from depression to spiritual cleanse I go to the ocean. I’ve healed many wounds there”; “Swimming with a pod of wild dolphins was a religious experience. The way they interacted with me and each other was kind, inquisitive, gentle, and protective.”	10.6%

The predominant theme, mentioned by 248 respondents (22%), concerned encounters with marine wildlife and sea creatures (e.g., dolphins, stingrays, sea turtles, fish, sea lions, sea birds, whales, orcas). Sample responses included “walking on the beach with my family at sunset and seeing a pod of dolphins jumping in and out of the water” or “seeing a shark in Bermuda.” Participants described these ocean life moments as especially memorable and emotional. Many focused on the thrill and joy of seeing marine creatures in their natural environment, such as “walking on the beach with my family at sunset and seeing a pod of dolphins jumping in and out of the water,” “snorkeling above a sea turtle,” or “seeing a shark in Bermuda.” For some, these ocean encounters were brief but memorable experiences of what lies below the oceans surface. Respondents frequently associated wildlife experiences with feelings of awe, gratitude, and a sense of privilege at being “allowed” into the marine environment. Several emphasized that “seeing animals up close” or watching them behave freely, rather than in captivity, changed how they thought about the ocean and marine species. Even when participants did not explicitly mention ocean conservation, their descriptions suggested that sharing the ocean with dolphins, whales, or sea turtles helped them see the ocean as alive and in need of care and protection.

Furthermore, a total of 184 participants (16%) described the ocean as a source of well-being–that is, as a calming and grounding place that provides tranquility and relief from stress. There was a positive sentiment toward marine experiences, frequently described by adjectives such as good and great. Likewise, some participants described their experiences with the ocean as relaxing (“My peaceful place was always to sit on a bluff about the ocean, breathe the air, listen to the waves and feel at one with the earth”), meditative (“Walking on the beach next to the ocean reminds me how small a part I am and what power the ocean has. I find it relaxing and comforting”), and calming (“When at the beach I was able to feel calm due to the sounds and view. It made me forget the troubles I was dealing with”). Collectively, these accounts describe the ocean as a refuge, a place where people can relax and reconnect with themselves and the world around them.

The third theme in the participants’ responses to the second question concerned the perception of the ocean as a source of adventure and recreation via the activities such as snorkeling, scuba diving, kayaking, and exploring tide pools for recreational fun. Overall, 160 respondents (14%) shared this view. For instance, one participant mentioned that “I kayaked surrounded by sea lions in Cabo San Lucas. They were interacting with us and playing with us. They were not at all threatening.” Another respondent said that, “While snorkeling, seeing the wildlife under the surface is always meaningful to me. Body surfing in ocean waves as well.” Yet another participant shared the following account: “Kayaking is important where I live. We have kept the Gualala River clean and free from junk. It’s so important for the river as it goes into the Pacific every day.” Together these narratives depict the ocean as a recreational area where adventure and fun foster a sense of connection to the marine environment and motivation to help keep the ocean healthy.

The fourth common theme mentioned by 148 respondents (13%) concerned shared experiences of spending time with family and friends. For many participants, the beach and ocean were beautiful places filled with memorable experiences. Representative responses in this theme included “Connecting and bonding with my kids and family while at the beach.” Several more extended quotes pertaining to this theme are quoted below.

I had a bonfire with friends at the beach, and the wind and the smell of the ocean and the feel of the sand reminded me that nature can help connect me with my physical being; just years of growing up on the beach and being with friends and family, I’ve always had a pull to the ocean, to see all the creatures God has created.

I have had many meaningful experiences at the beach and in the ocean. One of my favorites is when my cousin, aunt and I all went to Seaside, or, a beach we went to regularly, and we had a bonfire on the beach after playing in the water all day. We walked to haystack rock and ate some locally sourced clam chowder, we saw lots of marine life and just relaxed. At the bonfire, we talked about all our favorite memories of our family. The smell of the ocean and the sound of the waves are so peaceful. We also had a beachfront hotel room, and we love leaving the windows open to fall asleep to the sound of the waves.

Interestingly, several respondents mentioned important romantic moments, such as proposals, weddings, or walks with loved ones by the ocean (e.g., “My husband proposed to me at the beach because it is something that means a lot to both of us”). These accounts suggest that the ocean can function as a deeply meaningful relational space, an environment where commitments are made and relationships are celebrated. In this way, marine environments become places of special shared moments, fostering both attachment to loved ones and the ocean.

Another theme was ocean stewardship, along with environmental responsibility and respect, which was mentioned by 77 respondents (7%). Here, the participants referred to beach cleanups, protecting wildlife, and appreciating the importance of a clean ocean (i.e., “I helped a crab back to the water”; “I saved a starfish from dying”; “Watching a video of a large marine animal being rescued from shallow waters”). For many participants, ocean experiences reinforced the sense that “what we do matters,” and underscored a personal responsibility to keep the ocean and beaches clean to reduce harm to marine life. Even small actions such as moving a stranded animal or picking up litter, were described as moments that elicited understanding of human impacts on the ocean and their own role to care for it.

Finally, the ocean was reported as a source of feeling “one” with nature as well as a spiritual connection. This theme was particularly frequent in the responses from the surfer cohort. “One,” “oneness,” and spirituality were mentioned as meaningful experiences when in a marine environment (*N* = 53; 5%). For example, one participant said that, “the ocean has always been my element. In my darkest sadness from depression to spiritual cleanse, I go to the ocean. I’ve healed many wounds there.” Another participant noted that, “A spiritual sense of the ocean as my Mother.” A similar account was provided by another respondent: “I went to a beach, knelt down at the water’s edge and thanked the ocean for its beauty and immensity.” Taken together, these narratives describe the ocean as more than just a recreation setting but more importantly as a relational or sacred place that supports emotional healing and fosters a sense of connection to something larger than oneself.

Table 4 reports adjusted risk ratios with 95% CIs for the likelihood of mentioning each of the 7 themes related to meaningful ocean experiences and encounters by gender, age, ethnicity, and U.S. region. Female respondents were more likely to mention the themes of wildlife encounters/sea creatures (RR = 1.41, 95% CI [1.12, 1.77], *p* = 0.003) and well-being, calm, aesthetic appreciation (RR = 1.80, [1.36, 2.39], *p* < 0.001), and adventure and recreation (RR = 1.35, [1.00, 1.81], *p* = 0.047). Geographic regions in the US were also associated with several themes: residing in the South Atlantic was associated with more wildlife mentions (RR = 1.33, [1.02, 1.73], *p* = 0.037); living in the Mountain region was associated with more adventure mentions (RR = 1.63, [1.02, 2.60], *p* = 0.041); and living in the Middle Atlantic was associated with more mentions in the themes good/great (RR = 2.64, [2.00, 3.49], *p* < 0.001) and one/spirituality (RR = 2.92, [1.74, 4.93], *p* < 0.001). For family, friends, and social connections, younger adults (18–29) were more likely to mention this theme (RR = 1.42, [1.01, 2.00], *p* = 0.043). No covariates were significantly associated with stewardship, environmental responsibility and respect in the adjusted model.

**TABLE 4:** Adjustedrisk ratios for thematic mentions of meaningful ocean experiences by gender, age, ethnicity, and U.S.geographic region.

Theme	Significant predictors (RR [95% CI])	*P*-value
Wildlife encounters / sea creatures	Female: 1.406 [1.119, 1.766]	0.003
	*South Atlantic: 1.327 [1.018, 1.730]	0.037
Well-being calm, aesthetic appreciation	Female: 1.799 [1.356, 2.386]	<0.001
Adventure and recreation	Female: 1.348 [1.004, 1.810]	0.047
	*Mountain: 1.630 [1.020, 2.604]	0.041
Family, friend, and social connections	Age 18–29: 1.424 [1.012, 2.003]	0.043
Good/great	Male: 1.805 [1.334, 2.443]	<0.001
	*Middle Atlantic: 2.638 [1.995, 3.488]	<0.001
Stewardship, environmental responsibility and respect	No significant associations	–
One, spirituality	*Middle Atlantic: 2.925 [1.736, 4.928]	<0.001

*Mountain: Arizona; Colorado; Idaho; Montana; Nevada; New Mexico; Utah; Wyoming. *Middle Atlantic: New York; New Jersey; Pennsylvania. *South Atlantic: Delaware; Maryland; District of Columbia; Virginia; West Virginia; North Carolina; South Carolina; Georgia; Florida.

The geographic patterns observed in Table 4 suggest that the meanings people take from the ocean vary across the United States by regional coastlines, histories, and ways of using marine environments. Respondents living in the South Atlantic were more likely to mention wildlife and sea creatures, those in the Mountain region more often emphasized adventure and recreation, and participants in the Middle Atlantic highlighted “good/great” experiences and spirituality. These differences may reflect the opportunities that local coastlines and waterways afford, such as frequent wildlife sightings in some regions or culturally embedded uses of beaches and rivers for recreation, reflection, or spiritual practice. From an intervention perspective, these patterns point to the value of place-based and culturally grounded approaches to strengthening ocean connectedness and literacy. In regions, like the South Atlantic, where wildlife encounters are already a common feature of people’s experiences conservation campaigns might leverage charismatic marine species and everyday marine wildlife viewing as gateways to deeper understanding of marine ecosystems and threats. In areas where adventure and recreation are central messages could build on people’s attachment to paddling, snorkeling, or tide pool exploration to highlight ocean pollution pathways and shared responsibility for keeping upstream environments clean. In the Middle Atlantic, where respondents more often described the ocean as “good/great” and spiritually meaningful, interventions may resonate more that focus on gratitude, care for creation, and the ocean as a source of meaning and perspective.

The lack of significant demographic predictors for the stewardship theme suggests that concern and responsibility for the ocean are universally important by region, gender, or age group. Collectively, these findings indicate that the prominence of particular ocean meanings varies across gender, age, and region, but ocean stewardship is a wide spread concern. This pattern underscores the potential for ocean conservation campaign interventions that are tailored to local areas while recognizing that ocean stewardship is a widely shared value and concern.

## 4 Discussion

The results of the present study provide a nuanced account of how American adults understand the ocean, what they feel in marine environments, and how these experiences relate to stewardship and everyday behaviors. Rather than treating “ocean connectedness” as a single score, the themes that emerged, such as well-being and calm, wildlife encounters, adventure and recreation, social connection, interconnection of all life, general feelings that “life is good/great,” and spirituality/oneness, illustrate multiple ways in which people care and relate to marine environments. These findings help bridge work on blue spaces and mental health, nature connectedness, and pro-environmental behavior by grounding those constructs in concrete stories of what the ocean means to Americans. In this way, the study complements prior quantitative research on ocean connectedness and environmental identity by centering participants’ narratives about how the ocean shapes emotions, relationships, and ocean stewardship.

A central contribution of this study is the detailed description of the ocean as a source of well-being, calm, and emotional restoration. Participants described the ocean as an “everyday refuge” where they could reset, breathe more easily, and feel grounded again. These narratives align with growing evidence that blue spaces are associated to lower stress, improved mood, and enhanced psychological well-being (Fleming and Landrigan, 2024; White et al., 2021). The sensory qualities people reported, such as the sound of waves, salt air, light on the water, are also prominent in the blue health literature, as well as the biophilia hypothesis (Wilson, 1986). The present findings add texture to prior quantitative work and underscore that ocean connectedness is not only about identity or values, but also about how people regulate emotions and cope with stress in marine settings. For many respondents, the ocean was an accessible health resource supporting emotional well-being.

Encounters with marine wildlife formed another powerful pathway to ocean connection. Participants described encounters with dolphins, whales, sea turtles, sharks, seabirds, and other species with awe, privilege, and gratitude. This is consistent with research showing that direct experiences with wildlife can deepen emotional bonds with nature and motivate conservation behaviors (Pahl et al., 2017; Buchan, 2021; O’Halloran, 2025). The present study extends that work by highlighting how these marine encounters are often social (e.g., watching dolphins with family) and combine emotions aspects like awe, beauty and joy, and a sense of ocean stewardship. From a marine conservation perspective, these narratives suggest that carefully designed, ethical wildlife experiences can provide education, foster emotional connection, and ocean stewardship.

Adventure and recreation themes further illustrate how ocean connectedness is often built through embodied, physical engagement with the marine environment through snorkeling, surfing, kayaking, or exploring tide pools. These recreation activities are fun and deepen attachment and awareness of local marine environments. This resonates with research on place attachment and environmental identity, which suggests that repeated, meaningful activities in nature can make places feel like “home” and, in turn, support protective intentions and behaviors. The present findings reinforce the idea that ocean literacy and ocean conservation campaigns can meet people where they already find joy, such as on beach walks, in kayaks, or exploring tide pools. Building on ocean and beach experiences can lead to ocean stewardship actions.

The theme of interconnectedness points toward more cognitive and ecological ways that ocean connectedness may develop. Respondents described the ocean as the planet’s “heartbeat” and recognized its role in climate regulation, biodiversity, and human survival. This systems thinking echoes key ocean literacy principles and aligns with work showing that understanding ecological interdependence can support more pro-environmental attitudes and behaviors. Importantly, in many narratives this sense of interconnection was associated with stewardship. Acts such as returning stranded animals to the water, picking up litter, or participating in beach cleanups illustrate how awareness and emotional attachment can translate into small but meaningful ocean stewardship behaviors (Buchan, 2021; McKinley et al., 2023; O’Halloran, 2025). These findings suggest that ocean literacy efforts that explicitly connect the ocean’s life sustaining functions to everyday ocean actions may help people move from awareness to action.

Across themes, positive emotions played a central role. Participants described happiness, gratitude, awe, beauty, and joy as part of their most meaningful ocean experiences. This pattern is consistent with Thomas-Walters et al.’s (2023) conclusion that emotional engagement is a key driver of environmental behavior change. In the present study, awe at wildlife, joy in play, gratitude for beauty, and comfort in times of distress all appear as emotionally resonant and may led to ocean stewardship. Spiritual and “oneness” narratives extend this further, portraying the ocean as a sacred or healing presence, “my Mother,” a place for spiritual cleansing, or a site of deep gratitude. These findings suggest that ocean connectedness is not only experiential and cognitive, but also emotional, cultural, and spiritual, especially for certain groups such as surfers. Attending to this full spectrum of emotions, including spiritual and existential meanings, may be important when designing ocean conservation interventions.

The regression results demonstrate ocean connections are patterned across demographic groups and regions. Women in this sample were more likely to mention wildlife, well-being, and adventure themes; younger adults more often emphasized family, friends, and social connections; and regional patterns emerged for wildlife (South Atlantic), adventure (Mountain region), and spiritual/good/great themes (Middle Atlantic). These differences point toward place based and audience specific interventions for ocean stewardship. In regions where wildlife experiences are already salient, messages and programs that highlight charismatic species and local biodiversity may resonate strongly. In areas where recreation is central, building stewardship into paddling, snorkeling, or beach based activities may feel natural and relevant. Where spiritual and existential meanings dominate, interventions that speak to gratitude, care for creation, and the ocean as a source of meaning may be more compelling than purely technical or risk focused messages. At the same time, the absence of strong demographic predictors for ocean stewardship in the adjusted model suggests that concern for the ocean across all communities.

The qualitative study findings cannot establish causal pathways between specific kinds of ocean experiences, ocean connectedness, and pro-environmental behaviors. The cross-sectional study design focused on people who could recall at least one meaningful experience in a marine environment. As such, the study cannot determine whether direct encounters with the ocean are necessary or sufficient for ocean connectedness or stewardship, nor can it speak to people who have limited access to the ocean, feel indifferent toward it, or are scared of it. The study goal was to describe how people who already have some connection to the ocean talk about it, and to identify themes that may be useful for ocean conservation interventions and campaigns. Additionally, some people may develop strong ocean identities and stewardship motivations through mediated experiences such as ocean documentaries, social media, books, community activities, cultural or spiritual traditions even without frequent ocean visits. Future studies should include people with infrequent, neutral, or negative ocean experiences, to compare their narratives with those reported here.

## 5 Limitations and future directions

Several limitations should be considered when interpreting the study findings. First, the data are based on self-reported narratives from American adults and may not reflect ocean experiences in other cultural, socioeconomic, or geographic contexts. Replicating this work with coastal and inland communities in other countries, and with groups who differ by age, occupation, or cultural ties to the ocean, will help clarify which themes are more universal and which are context-specific. Second, the wording of the two open-response questions explicitly invited participants to reflect on what the ocean “teaches about life” and to describe “meaningful” experiences. This framing likely weighted the dataset toward respondents with positive ocean perspectives. People with indifferent, weak, or predominantly negative relationships to the ocean may be underrepresented. Future research should recruit individuals who rarely visit the coast, feel disconnected from the ocean, or hold ambivalent or negative views, and use alternative prompts that do not presuppose meaning or life lessons. Third, themes were developed and applied by a single coder. Although the analysis followed a reflexive thematic approach with three independent coding passes, within-coder checks, and an audit trail documenting code decisions, the use of one coder limits the ability to assess reliability and increases the risk of interpretive bias. Future studies should involve multiple coders, use structured training procedures, and report inter coder reliability statistics to strengthen confidence in the thematic structure. Fourth, a substantial minority of participants provided only one word responses. These minimal answers constrained interpretive depth and may reflect less elaborated ocean memories, lower importance of the ocean in their lives, or pragmatic survey factors such as time fatigue. While they may signal weaker ocean relationships for some respondents, the current design cannot determine this. Future studies with follow-up interviews or mixed-methods designs could discern meanings behind these brief responses. Finally, the qualitative, cross-sectional nature of this study precludes causal inference between ocean experiences, connectedness, and pro-environmental behaviors. Longitudinal and experimental designs are needed to test whether changes in specific types of ocean experiences (e.g., wildlife encounters, family-based recreation, spiritual practices) lead to measurable changes in ocean connectedness and ocean stewardship over time.

Despite these limitations, the present findings offer practical insights for those working at the intersection of ocean conservation, education, and health. The themes identified here suggest that interventions may be more effective when they intentionally cultivate multiple pathways to ocean connectedness: emotional (awe, joy, gratitude), relational (family, friends, romantic bonds), experiential (recreation and adventure), cognitive (understanding interconnection and ocean literacy principles), and spiritual or existential (meaning, refuge, oneness). Programs that combine direct experiences (e.g., local coasts, rivers that connect to the ocean, immersive experiences) with storytelling and reflection may help people to understand and care about the marine environment.

In conclusion, this qualitative study illustrates that, for many American adults, the marine environment is experienced as a source of beauty, well-being, joy, wonder, adventure, community, spiritual meaning, and stewardship. Although these findings cannot establish that particular forms of contact are necessary for ocean connectedness or conservation behavior, they do offer insights for ocean literacy, public health, and conservation efforts to support oceans and human health and well-being. Ocean conservation and education initiatives can draw on these themes to promote positive ocean experiences, facilitate meaningful wildlife encounters, and encourage family, friend, and community engagement for ocean stewardship.

## Data Availability

The raw data supporting the conclusions of this article will be made available by the authors, without undue reservation.

## Footnotes

1
https://www.surveymonkey.com/
